# The Efficacy of Transcranial Current Stimulation Techniques to Modulate Resting-State EEG, to Affect Vigilance and to Promote Sleepiness

**DOI:** 10.3390/brainsci8070137

**Published:** 2018-07-20

**Authors:** Ludovica Annarumma, Aurora D’Atri, Valentina Alfonsi, Luigi De Gennaro

**Affiliations:** Department of Psychology, University of Rome Sapienza, 00185 Rome, Italy; ludovica.annarumma@uniroma1.it (L.A.); aurora.datri@uniroma1.it (A.D.); valentina.alfonsi@uniroma1.it (V.A.)

**Keywords:** transcranial Current Stimulation (tCS), transcranial Direct Current Stimulation (tDCS), transcranial Alternating Current Stimulation (tACS), oscillatory transcranial Direct Current Stimulation (osc-tDCS), EEG, sleep, sleepiness, vigilance, sleep disorder, clinical application

## Abstract

Transcranial Current Stimulations (tCSs) are non-invasive brain stimulation techniques which modulate cortical excitability and spontaneous brain activity by the application of weak electric currents through the scalp, in a safe, economic, and well-tolerated manner. The direction of the cortical effects mainly depend on the polarity and the waveform of the applied current. The aim of the present work is to provide a broad overview of recent studies in which tCS has been applied to modulate sleepiness, sleep, and vigilance, evaluating the efficacy of different stimulation techniques and protocols. In recent years, there has been renewed interest in these stimulations and their ability to affect arousal and sleep dynamics. Furthermore, we critically review works that, by means of stimulating sleep/vigilance patterns, in the sense of enhancing or disrupting them, intended to ameliorate several clinical conditions. The examined literature shows the efficacy of tCSs in modulating sleep and arousal pattern, likely acting on the top-down pathway of sleep regulation. Finally, we discuss the potential application in clinical settings of this neuromodulatory technique as a therapeutic tool for pathological conditions characterized by alterations in sleep and arousal domains and for sleep disorders per se.

## 1. Introduction

Transcranial Current Stimulations (tCSs) are a family of neuromodulatory techniques that, via the application of low-intensity current on the scalp, can modify cortical activity influencing motor, emotional and cognitive functions [1,2]. tCSs are defined as Non-Invasive Brain Stimulations in that they act on brain physiology but do not involve surgical implantations (e.g., Deep Brain Stimulation) [3]. As tCSs show small side effects, they offer a viable and promising tool to modulate cortical activity in a quite predictable, safe, economic, and non-invasive manner.

Recently, tCSs have been used during sleep or during quiet or active wake to influence sleep and arousal patterns. The sleep-wake regulation mechanism has still to be fully understood. For many years, the prevalent view considered a bottom-up direction of sleep control from subcortical to cortical structures. Specifically, the sleep-regulating circadian and homeostatic signals are integrated into the Ascending Reticular Activating System (ARAS), originating in the brainstem [4]; this system projects to the thalamus and the cortex by two distinct pathways. Therefore, high activity in the ARAS forms a wake-promoting system, whereas low activity is necessary for the occurrence of sleep [5]. In more recent years, the top-down component in the control of sleep regulation has been highlighted. Evidence from animal studies shows that slow oscillations are generated by a complex interaction between neocortex and thalamus [5]; neurons of IV-VI layers represent the primary oscillator of the cortico-thalamo-cortical feedback loop [6,7,8,9]. These populations induce synchronized rhythmic fluctuation of membrane potential in the sense of hyperpolarized Down state and depolarized Up state. The depolarization of the Up state flows as a traveling wave to cortical layers and areas and the thalamus [10]. This loop seems to be orchestrated by a subgroup of GABAergic interneurons. The activation of reticular thalamic neurons induces a bisynaptic inhibition of thalamo-cortical neurons, making them the intermediary between cortico-thalamic and thalamo-cortical neurons [11,12]. The inhibition of thalamo-cortical neurons induces an excitatory thalamo-cortical signal, which closes the loop [6,13]. The cortical component (top-down pathway), so far neglected, plays a pivotal role in sleep/arousal regulation.

The new scenario of top-down control of sleep and arousal regulation sheds light on the possibility of modulating these patterns through transcranial stimulation of the cortical component of the cortico-thalamic feedback loop.

Here, we explore this issue with specific reference to stimulation with direct current, i.e., transcranial Direct Current Stimulation (tDCS), and stimulation with time-varying current, i.e., transcranial Alternating Current Stimulation (tACS) and oscillatory tDCS (osc-tDCS). After a brief discussion on the mechanisms of action, we examine evidence in support of tCS efficiency in modulating electroencephalographic (EEG) rhythms.

Purposely, we aim to review critically the studies in the literature that applied tDCS, tACS, and osc-tDCS to regulate sleep and vigilance patterns. Specifically, the spotlight of this work is to provide a systematic overview of the studies that aimed to increase Slow-Wave Sleep (SWS) or sleepiness and, conversely, of works that aimed to enhance vigilance and to reduce sleepiness levels, analyzing electrophysiological and behavioral evidence in healthy subjects. Finally, we analyze the possible application of tCSs on several clinical conditions, including psychiatric or organic disturbances and sleep disorders.

## 2. Features, Mechanisms and tCSs Efficacy in Modulating Cortical Rhythm

Regarding the ability of these paradigms of stimulation to modulate cortical activity, it is important to consider that different techniques take advantage of different mechanisms of action. Transcranial Direct Current Stimulations (tDCS) devices comprise a battery-powered current generator that delivers a constant electrical current flow via two or more surface electrodes (cathode and anode) placed over the scalp. During the stimulation, the current flows from the anode to the cathode [14] influencing the excitability of the neuronal populations (pyramidal cells), underlying the stimulation electrodes. Specifically, the external current field forces the arrangements of intracellular ions, altering the distribution of the internal charge and modifying the resting transmembrane potential [15]. Generally speaking, the direction of the induced effects mainly depends on the polarity of stimulation: anodal stimulation induces a sub-threshold depolarization [15] resulting in increases of the average firing rate [16], whereas cathodal stimulation induces the opposite effects. On the one hand, numerous studies modeling tCSs-induced intracranial current flow have shown that a large amount of the applied current is short-circuited by the skin [17,18,19]. In this sense, the magnitude of the current density, manipulated through different shape/size of the electrode assembly and current intensity applied result pivotal in determining the proportion of current that arrives intracranially [19]. On the other hand, the complexity of brain morphology implies that it is the direction of the current relative to the orientation of the target neural pathway that determines the effect of stimulation [20]. In particular, a current with a soma-dendrite direction causes hyperpolarization of the soma and suppresses the action potentials of the neural population; on the contrary, a dendrite-soma direction of the current induces depolarization of the soma [21,22]. Accordingly, stimulation with the same polarity could induce different effects in function of the electrode placement since the direction and the strength of the effects depend on the relative position of anode and cathode with respect to the direction of the pyramidal cells in the stimulated area [23]. Furthermore, it is worth noting that the inter-electrode distance (i.e., the cephalic or extracephalic position of the “return” electrode), the inhibitory or excitatory connections of the target area, as well as the whole set of cortical and subcortical regions through which the current flows, affect the amplitude of the local and global cortical effects of the stimulation [23,24].

At macroscopic level, the modulation of the firing rate by tDCS results in changes of cortical activity in specific frequency bands, as measured by EEG. Summarizing the results from the different EEG studies with tDCS, anodal stimulation has been linked to increases of beta power [25,26,27,28] and reduction of delta, theta, and alpha power [26,27,29,30], while cathodal stimulation results in the reduction of beta and gamma power [25], and the increase of theta and delta power [31]. From a physiological point of view, EEG patterns characterized by higher power in high-frequency bands paralleled by lower power at low frequencies, as with those induced by anodal tDCS, are associated with cortical desynchronization and alert state.

tACS and osc-tDCS share the modality of the current injection with tDCS, but they stimulate with a time-varying current intensity. In tACS protocols, an alternating current is applied, and, in most cases, the current has a sinusoidal wave form. The transcranial application of alternating current induces periodic shifts in the transmembrane potential, alternating depolarizing and hyperpolarizing effects as a function of the phase shift. In other words, the stimulation at a given frequency drives the cortical network to oscillate at the stimulation frequency with a greater amplitude (resonance phenomenon). In osc-tDCS, tDCS and tACS are combined, resulting in a polarized stimulation that benefits from both the effect of direct current on cortical excitability and the synchronizing effect of the alternating current on the ongoing brain activity. According to Bergman [32], the mechanism by which osc-tDCS exerts its effects may depend, as with tACS, on the shifting the membrane potential repeatedly back and forth in an oscillating manner.

At cortical level, the synchronization of the firing rate with the variations of the electric field results in increases of the EEG spectral power at the frequency of the applied current. For example, theta-tACS during wakefulness can enhance the theta activity [33,34]. Voss and coworkers found out that stimulating the fronto-temporal area at 40 Hz and, to a lesser degree, at 25 Hz, during Rapid Eye Movement (REM) sleep, leads to local increase in gamma activity [35]. Lustenberger and colleagues [36] applied a feedback-controlled 12 Hz tACS on frontal areas during non-REM (NREM) sleep, stimulating only when spindles occurred. This approach selectively enhanced cortical synchronization in spindle frequency range, without altering the sleep architecture.

It should be underlined that the efficacy in inducing resonance phenomena by means of time-varying stimulations critically depend on the physiological rhythmic activity spontaneously expressed by the target area. For instance, in an eye-closed condition, characterized by alpha activity, alpha-tACS can induce resonance between frequency of stimulation and ongoing cerebral activity, whereas beta-tACS cannot [37]. Furthermore, applying alpha-tACS to the areas that preferentially oscillate at this frequency (such as the occipital cortex [38]) induces greater increases of EEG power at the given frequency than stimulating other regions (i.e., temporal cortex [18]).

The efficacy of osc-tDCS has been shown in different studies [39,40] that used it to boost or disrupt specific cerebral activity. Similar to tACS, the consistency between the stimulation frequency and the ongoing cortical activity in the target area plays a fundamental role in determining the stimulation outcomes. For instance, a stimulation oscillating at 0.75 Hz was able to increase slow oscillations (0.5–1 Hz), when applied during the first part of the night [39]—a period rich in slow waves—whereas stimulating at 5 Hz (theta range) did not increase but reduced the slow oscillations. On the other hand, applying this frequency of stimulation (5 Hz) during REM sleep induces enhancement of the gamma activity [40]. This result might seem contradictory since no increase in the theta activity was reported; however, boosting the theta rhythm, which is inherent to REM stage [41,42,43] but not extremely expressed at the moment of stimulation, might have synchronized gamma band activity via cross-frequency functional coupling mechanism [40]. Interestingly, D’Atri and coworkers [44] applied 5 Hz and 0.8 Hz oscillatory stimulations in a resting wake period, characterized by a more rapid activity than SWS, and found a frequency-specific enhancement of theta oscillations along with cross-frequency increases in slow oscillations induced by the 5 Hz stimulation compared to the 0.8 Hz stimulation [44]. Regarding the role of the stimulation polarity, empirical findings seem to support a more robust efficacy of the anodal oscillatory stimulation compared to the cathodal one [39,40,45]. This is directly shown in a study that compared the two oscillating stimulations (cathodal vs. anodal) showing a larger effectiveness of the anodal one [46].

Therefore, all these results show the capability of these types of techniques of modulating cortical activity and, moreover, stress the importance of setting the appropriate stimulation parameters, such as polarity and the frequency, taking into account the brain state activity, and choosing the appropriate area target of stimulation, for reaching specific goals.

## 3. Promoting Sleepiness

From an electrophysiological point of view, promoting sleepiness, sleep, or a “good-quality sleep” can be referred in modulations of cortical activity resulting in enhancements of theta and alpha activity during the wake or Slow-Wave Activity (SWA, 0.5–4 Hz) during sleep.

Historically, the application of an external current to induce sleep-like states goes back to the beginning of the 20th century [47]: electrosleep therapy used a pulsating direct current for 30 to 120 min, with the electrodes attached to eyes and mastoids [48]. Since the studies using this technique are dated, most of them lack methodological rigor (e.g., no control with sham condition or no EEG measures) and sometimes show contrasting results that may depend on electrical characteristic of the device used or other methodological issues such as, for example, the duration of stimulation [49]. Anyway, even if a large amount of electrosleep studies applied the stimulation on clinical populations (see Section 5), this technique resulted in improvement of sleep also in normal subjects [50]. From the first promising evidence reporting effects on sleep, anxiety, and depression [48], there was a growing interest in the study of these stimulations that leads to the recent development of tCSs. Accordingly, in the last few decades, many studies used tCSs during sleep or during quiet or active wake to enhance SWA, affect sleep propensity, or to ameliorate qualitative and quantitative sleep parameters.

Most of the studies available in the literature applied the stimulation during NREM sleep stage 2 or SWS, to boost the SWA [39,51,52,53,54,55,56,57,58]. According to empirical evidence pointing out that frontal areas are the first regions that “fall asleep” [59,60,61], and that SWA is predominant in frontal areas during sleep [62,63], these studies stimulated frontal regions and obtained an enhancement of SWA spectral power, both in normal subjects [39,51,52,53,54,55] and in patients [56,57,58].

Anodal oscillatory stimulation at 0.75 Hz (slow osc-tDCS) in frontal areas during sleep stage 2 of a diurnal nap [51,52,53] or during nocturnal sleep [39,54,56,57,58] can induce a frequency-specific enhancement of SWA, compared to sham, with little or no disturbing effects induced by stimulation. Marshall and colleagues [39] applied the stimulation on fronto-lateral locations, in five 5-min blocks with 1 min free of stimulation interval, after 4 min of stage 2, a period in which sleep becomes deeper and cerebral activity slows down, evolving into SWS. The stimulation locally enhanced the EEG power in the slow oscillation range (0.5–1 Hz) as revealed by the analysis of the 1-min intervals. This effect was frequency-specific, since only 0.75 Hz stimulation was effective in enhancing SWA, whereas the 5 Hz stimulation reduced SWA [39].

Among the mentioned studies, some were conducted on elderly populations [52,53,54]. These studies applied oscillatory stimulation at 0.75 Hz on the frontal areas, delivered in five blocks, each lasting 5 min, with 1 min [52] or 1 min and 40 s [53,54] intervals free of stimulation, with the current intensity varying between 0 and 260 µA. Since sleep in elderly is disrupted, fragmented, and contains less SWS, the enhancement of slow oscillatory activity induced by slow oscillatory-tDCS in this population might be considered as a relevant finding, supporting the feasibility to ameliorate sleep quality by transcranial stimulations. Besides the positive and clear effect on sleep, an enhancement of SWS by slow oscillatory-tDCS in older subjects could also have beneficial effects on the physiological cognitive decline observed in this population, since the reduction of SWS has been associated with deterioration of different cognitive functions, such as memory [53] (see Section 5). Other evidence in older adults shows contrasting results [64,65]; for example, the same oscillatory stimulation (0.75 Hz) did not induce an enhancement either in sleep parameters or sleep-associated memory task. Differences in the experimental protocols (for example, stimulation procedures) and features of elderly sleep may account for the different results. For instance, Eggert and coworkers [64] applied a ramping period at the beginning and at the end of each stimulation interval to reduce skin sensation; as observed by Ladenbauer and colleagues [53], this ramping mode might have prevented the short-lasting stimulation-dependent entrainment of slow oscillation activity. Moreover, in this study the stimulus-free periods were not controlled and because elderly sleep is lighter and fragmented, it is thus possible that the stimulation did not occur in stage 2, but, instead, in lighter stages, preventing the resonance phenomenon between the ongoing activity and stimulation frequency.

In a very recent study, Sheng and coworkers [66] applied at frontal location an anodal high-definition-tDCS to improve sleep in the elderly and found an effective enhancement of sleep duration and a reported feeling of improved sleep.

Since evidence regarding older adults is conflicting, to evaluate the efficiency of tCSs on this specific population, future studies are needed.

We have already discussed the efficacy of 0.75 Hz stimulation in boosting SWA when it is applied during NREM sleep. When the same stimulation is applied during a wake period, one could expect a slightly different outcome, since during wakefulness our brain shows an EEG pattern of activity characterized by higher frequency waves with a smaller amplitude, compared to sleep. An oscillatory stimulation at 0.75 Hz in frontal areas during quiet wake (current intensity range 0–260 µA) enhanced slow-frequency (0.4–1.2 Hz) EEG activity in the area closest to the stimulation [45]. Additionally, 0.75 Hz anodal osc-tDCS increased also the theta activity, across electrode sites, showing the capability of this specific stimulation in inducing not only frequency-specific effects (the delta enhancement) but also cross-frequency outcomes (the theta increase). Since resting-state EEG is characterized by alpha and theta activity (the latter rhythm considered as a marker of sleepiness) it is reasonable to wonder if theta-tDCS (5 Hz) could induce a larger resonance effect than 0.75 Hz tDCS, also affecting the sleepiness level. In line with this, D’Atri and coworkers [46] compared these two frequencies of stimulation and found a post-stimulation significant enhancement of delta and theta activity after anodal stimulation at 5 Hz, compared to 0.75 Hz stimulation, combined to an increase of subjective sleepiness.

Moreover, in a recent sham-controlled study, D’Atri and coworkers [67] applied bilateral theta-tACS (5 Hz) at a fronto-temporal location for 10 min, during resting state, and found a posterior enhancement in theta power and a centro-frontal increase in alpha activity in the post-stimulation EEG, consistent with the increase of the sleep propensity after the stimulation. Interestingly, this effect was not only restricted to the specific bin corresponding to the stimulation frequency, but, instead, involved all the theta frequency band, as well as the alpha band with a peak at the 10 Hz bin, showing not only the entrainment of cortical oscillation, but perhaps also a more general synchronizing effect induced by the alternating stimulation [67].

All the aforementioned studies included a sham stimulation; the comparison with a control condition is fundamental to control for the placebo effect and to directly attribute the outcome to the stimulation rather than to other factors, such as the experimental procedure itself. Since only in very rare cases, the participants recognized the active stimulation (e.g., two subjects on a total sample of 18 in [53]), applying the stimulation for few seconds and then slowly ramping down the current intensity is shown to be an efficacious control condition [68].

In summary, all the aforementioned studies show not only the capability of these techniques to carry out a frequency-specific and cross-frequency modulation of cortical activity, but also provide evidence in support of tCS efficacy in inducing sleepiness and in boosting sleep and improving sleep quality, as assessed by electrophysiological and subjective measurement.

## 4. Promoting Vigilance

According to the notion of affecting synchronization of EEG by stimulation with slow-frequency tCS (i.e., promoting sleepiness), the modulation of cortical activity by reducing slow-frequency activity along with enhancing faster frequencies should boost the vigilance levels. Even if anodal tDCS seems able to induce this EEG pattern [25,26,27,28,29,30], evidence directly linking this cortical effect to the increase in vigilance levels are still scarce. Indeed, the studies applying tCS to increase vigilance that evaluated, besides standard vigilance measures, also quantitative EEG as outcome measures are quite exiguous. Nevertheless, the results from these few EEG studies seem to support tCS efficiency in promoting an EEG pattern of cortical arousal.

In a pilot study, applying a frontal tACS with a stimulation frequency of 30 Hz (10 min of stimulation; intensity 600 µA) had a sort of “sleepiness-preventing-effect” on the participants [69]. The control sham condition was associated with an increased self-reported sleepiness together with enhancement of delta power, while 30 Hz tACS appeared to block these physiological processes. A weak increase of the gamma power was also observed after 30-Hz tACS. These effects are consistent with a cortical activating effect of the stimulation. Further evidence supporting the tCS efficacy in reducing the sleep propensity is found in a study that applied a stimulation with direct current. Frase and colleagues applied a bilateral frontal anodal tDCS in healthy subjects (two blocks of 13-min stimulation; 1 mA) prior to sleep and found a significant reduction in total sleep time combined with an increase of intra-sleep-wake time and a decrease in sleep efficiency in the second part of the night. [70]. These macrostructural changes point to a much less deep and stable sleep after stimulation, suggesting a reduction of the physiological sleep pressure as a consequence of stimulation. Furthermore, a significant enhancement in the gamma activity was still present in the morning EEG, indirectly supporting this interpretation [70].

It is worth noting that the larger part of the studies mainly focused on behavioral measures of the stimulation efficacy in contrasting vigilance decrement during prolonged attentional tasks. Vigilance decrement is a phenomenon that occurs as time-on-task increases: the ability to detect targets decreases and the reaction times in stimuli detection also increase. The stimulation with direct current can reduce the decrement of vigilance, in terms of improvement of stimuli detection [71], if the current is applied during the task at 1 mA, for 10 min at frontal locations. Furthermore, frontal anodal tDCS (30 min of stimulation; 2 mA) appears to counterbalance the detrimental effect on vigilance induced by sleep deprivation more efficiently than caffeine [72,73]. Additionally, some indications suggest that the stimulation prevents vigilance decrement associated with sleep deprivation and induces an enhancement in self-reported fatigue, drowsiness, energy, mood, attention, and vigilance [72,73]. Interestingly, these studies found that the stimulation had stronger and more lasting outcomes compared to the consumption of caffeine gum, as also revealed by the Psychomotor Vigilance Task (PVT), a reaction time measure representing a behavioral index of sleepiness and vigilance [74]. These results are in line with others that found reductions of reaction times along with improvements in the performance of attentional tasks when frontal anodal tDCS (10 min of stimulation; 1 mA) was applied during the task [75].

Despite the small number of studies, the available data support the notion that tCS with anodal direct current or high-frequency tACS, compared to sham conditions, are techniques capable of enhancing the vigilance levels and/or to reduce sleepiness. It is necessary to consider that almost all the studies mentioned applied anodal tDCS to induce the intended effect; this is due to the specific facilitating effect on the firing rate exerted by this protocol. Moreover, the larger part of the studies only collected behavioral measures, and so future works should fill this gap, to elucidate the physiological mechanism underlying the activating effect of these stimulation protocols.

## 5. Clinical Applications

Recently, some clinical settings have begun to take advantage of the possibility offered by tCS techniques of modulating cortical activity in a quite predictive manner, applying them as complementary or alternative treatment for several disorders, e.g., Parkinson’s disease, pain, epilepsy, depression, and schizophrenia [76].

In the 1960s, several researchers applied electrosleep therapy, then called Cranial Electroteraphy Stimulation, to treat insomnia. Empirical evidence regarding the efficacy of this technique is not so robust and often shows methodological weakness. Nevertheless, in many studies this approach resulted in improvement of sleep disturbances (e.g., [77]). For instance, Cartwright and colleagues [78] applied this stimulation in insomniac patients and found a reduced sleep latency, a decreased awake time in bed, decreased duration of NREM sleep stage 1, an increase of NREM sleep stage 4 and increased percentage of delta activity. Interestingly, the stimulation benefits lasted for a long period (2 years) after the end of treatment [78].

Recently, only one study used tCS’s ability to promote sleep to treat sleep disorders involving difficulty in falling asleep or maintaining a stable sleep as in insomnia. Saebipour and coworkers [79] applied a frontal stimulation at 0.75 Hz during stage 2 in a population of insomniac patients; this protocol (five 5-min blocks of stimulation; current reaching 260 µA) appears to ameliorate sleep quality in direction of an increase of stage 3 duration, a decrease stage 1 duration, and a decrease of wake time after sleep onset. Conversely, several studies applied tCS to treat secondary sleep symptoms in different clinical conditions. Anodal tDCS during a wake period can improve the quality of subsequent sleep [80,81], as assessed by higher scores on the Pittsburgh Sleep Quality Index (PSQI), in post-polio syndrome [80] and euthymic bipolarism (if the cathode is placed on the cerebellum) [81], and to improve sleep efficiency and to decrease arousals in fibromyalgia [82]. Moreover, maybe as a consequence of the sleep improvement, the stimulations also induced ameliorations in several aspects of the quality of life (physical functioning, role physical, vitality, social functioning, and role emotional) in post-polio syndrome patients, and improvement of pain levels in fibromyalgia.

Another line of research tried to promote vigilance and/or decrease daytime sleepiness. Galbiati and coworkers [83] applied frontal anodal tDCS in hyperinsomniac patients for a period of 4 weeks (three stimulation sessions per week) and found a reduction in daily sleepiness as measured by Epworth Sleepiness Scale (ESS). This effect lasted for two weeks after the end of treatment. Contingently, the reduction of sleepiness was also confirmed by a decrease of self-reported sleepiness, as measured by a visual analog scale, and a reduction of reaction times in an attentional tasks (modified version of the Attentional Network Task, ANT), as objective measure. This is in line with another study in which tDCS is effective in contrasting the vigilance decrement in a population on Multiple Sclerosis patients [84]. Even if the results obtained by Galbiati and coworkers [83] are interesting and promising, the lack of a placebo control condition represents a limitation of the study. However, additional evidence in support of tDCS efficacy in enhancing vigilance in sleep disorders comes from a single case study on a hypersomniac patient: bi-frontal anodal tDCS (two blocks of 13-min stimulation, separated by 20 min stimulation interval; 2 mA) was indeed capable, compared to sham, of enhancing objective vigilance (PVT), subjective vigilance (visual analog scale) and to decrease the duration of daytime sleep (self-report measures) [85]. Conversely, a study that aimed to ameliorate vigilance, reduce daytime sleepiness and fatigue in Parkinson’s disease, applied anodal tDCS and obtained improvement to fatigue, but no effects were found on sleepiness, as measured by ESS [86]. The lack of effect observed in this study might derive from the specific altered sleep patterns in these patients.

Interestingly, according to the fundamental role of sleep for cognitive functioning (e.g., [56]), the possibility of influencing cortical activity during sleep has been used to improve cognitive symptoms linked to altered processes during sleep, in different pathological conditions. The enhancement in normal subjects of SWA induced by osc-tDCS at 0.75 Hz during sleep significantly improves performance in a memory task after sleep [39]; conversely, disrupting SWS by 5 Hz tDCS has a detrimental effect on memory performance [40]. The memory potentiation by the SWA boost has clear and important applications in clinical conditions suffering from memory complaints. Indeed, Mild Cognitive Impairment (MCI) patients can take advantage of these stimulation protocols. MCI is a clinical condition often evolving in Alzheimer disease (AD) [58]. The core symptoms in AD involve deficits in the memory domain [87], but these patients also report sleep impairments [88] that have been already documented in the MCI condition [89]. Ladenbauer and coworkers [58] found an increase in sleep slow oscillations (0.5–1 Hz) by means of frontal 0.75 Hz osc-tDCS and this improvement alleviated the memory impairment in participants with MCI, as indexed by a better performance in the visual recognition task.

tCS also alleviated the memory impairment in Attention-Deficit and Hyperactivity Disorder (ADHD). Recent evidence pointed to an impairment of sleep pattern, in particular to a dysregulation of slow oscillations. Indeed, boosting SWA with frontal 0.75 Hz osc-tDCS induces an enhancement in memory consolidation processes presumably associated to sleep in these patients [57]. Furthermore, as ADHD children have impaired executive functioning, the improvement of SWA employing frontal tDCS at 0.75 Hz had a positive effect on both slow oscillatory activity of sleep and behavioral inhibition [56]: <1 Hz slow oscillations increased as consequence of stimulation, and this was paralleled by a higher behavioral inhibition.

Del Felice and coworkers [90] took advantage of the memory-boosting effect of osc-tDCS at 0.75 Hz to improve memory alterations due to temporal lobe epilepsy. The stimulation (fronto-temporal locations) enhanced slow spindle generator current density; this result was accompanied by an improvement in memory performance; therefore, the authors linked the improved memory performance to the transcranial modulation of cortical source generator of slow spindles.

Even if the larger part of the discussed studies aimed to improve sleep alterations in several clinical conditions to ameliorate other symptoms of the specific disease, the evidence supports anyway the notion that tCSs can induce effects, not only on sleep parameters in normal subjects but also in sleep alterations associated with several diseases. The paucity of evidence regarding the application of transcranial technique on sleep disturbance is, however, accompanied by promising data [79,83,85]. In light of this, even if these results should be cautiously considered as preliminary, and therefore, further research is needed, they suggest the feasibility of tCS to improve sleep/vigilance disturbance, as a complementary or alternative treatment of sleep and arousal disorders.

## 6. Conclusions

Even if the use of tCS techniques began over a century ago, there has been a recent renewed interest in these protocols, and a fascinating application regards the use in basic and clinical settings, as complementary or alternative treatment of several disorders such as schizophrenia, depression, and epilepsy. Interestingly, some of these studies targeted sleep or vigilance patterns to induce improvement in the specific disease, through symptoms’ reduction.

In the light of the examined literature, on the one side, we have found how an oscillatory stimulation at 0.75 Hz on frontal areas during NREM sleep seems to be the most effective protocol capable of enhancing SWS, whereas a stimulation at 5 Hz during resting state is the suitable method for inducing and increasing sleepiness. The frequency-dependency of these outcomes is likely linked to the mechanism of resonance underlying the stimulation action. During NREM, the application of an external field within the frequency of the ongoing cerebral activity (SWS) allows the entrainment of cortical slow oscillations, while the increase of slow-frequency activity during wake seems associated with a stimulation frequency in the theta range, consistent with the physiological meanings of waking theta power as a sleepiness index. Moreover, the appropriate choice of the target area to modulate the sleep propensity appears to be the frontal regions, consistently with their pivotal role in sleep onset [59,60,61], and in slow-wave sleep [63]. In other words, we can look at the transcranial induction of cortical synchronization within slow frequency ranges in anterior areas as the effective external manipulation of the top-down component of the sleep control. On the other hand, when the aim is the enhancement of vigilance levels or the reduction of daily sleepiness, the application of direct current with anodal polarity seems to be the most efficacious protocol. According to facilitating effect of the anodal tDCS on firing rate, the stimulation with anodal polarity enhances cortical activation, inducing cortical desynchronization and an alert state. Unfortunately, this research area is less investigated, and only few of these studies provided electrophysiological evidence, which is necessary to link the observed self-reported or behavioral outcomes directly to the actual manipulation of cortical rhythms by tCSs. Future studies should expand this research area, providing electrophysiological evidence, combined with subjective and behavioral measurements. Nevertheless, the existing data provide some support to the efficacy of tCSs in enhancing vigilance/reducing sleepiness. Given the implications that the level of sleepiness has for the individual’s current and near-term ability to perform operationally relevant tasks safely and efficiently, a tool that is able to manipulate the arousal level (increase and decrease) in healthy subjects could have high social impact.

Fascinatingly, clinical applications and, in particular, those studies that targeted sleep or arousal pattern to ameliorate specific sleep disorders, are promising, with a potentially high impact as a novel treatment for specific diseases. Their relevance also extends to basic research. Firstly, the mechanisms of sleep and arousal regulation are not entirely understood; hence, altering sleep/arousal pattern through stimulation of top-down pathway might shed light on a deeper knowledge of sleep modulating mechanisms. Secondly, the origin of these disorders might involve functional alterations in the feedback loop between cortical and subcortical components of the sleep-wake cycle regulation [5]; the modulation of the top-down component of this loop by tCS could restore the altered network. Even if only a few preliminary studies [79,83,85] tested this possibility, nevertheless they support the possibility of using tCS as a promising tool for treating sleep disorders.

Finally, since sleep complaints are common features of many neuropsychiatric conditions, the potentially high impact of using tCSs as a novel treatment also involves sleep complaints secondary to neuropsychiatric conditions, such as ADHD [56] or MCI [58], and potentiation of cognitive functions depending on better sleep quality.

Future work should explore this area, using larger samples, testing different protocols, and setting appropriate parameters of stimulations. Further investigation of the possibility of tCSs as treatment for sleep disorder might bring remarkable advances in research, especially in clinical fields, providing a possible treatment, complementary or alternative to drugs, since prolonged drug intake could result in serious side effects, the development of tolerance, sensibilization and the rebound effect [91].
